# Evaluating the Validity of Current Mainstream Wearable Devices in Fitness Tracking Under Various Physical Activities: Comparative Study

**DOI:** 10.2196/mhealth.9754

**Published:** 2018-04-12

**Authors:** Junqing Xie, Dong Wen, Lizhong Liang, Yuxi Jia, Li Gao, Jianbo Lei

**Affiliations:** ^1^ Department of Epidemiology and Biostatistics School of Public Health Peking University Health Science Center Beijing China; ^2^ Center for Medical Informatics Peking University Beijing China; ^3^ The Affiliated Hospital of Guangdong Medical University Zhanjiang China; ^4^ Department of Medical Informatics School of Public Health Jilin University Changchun China; ^5^ School of Stomatology Peking University Beijing China; ^6^ School of Medical Informatics and Engineering Southwest Medical University Luzhou China

**Keywords:** wearable electronic devices, fitness trackers, data accuracy, physical activity

## Abstract

**Background:**

Wearable devices have attracted much attention from the market in recent years for their fitness monitoring and other health-related metrics; however, the accuracy of fitness tracking results still plays a major role in health promotion.

**Objective:**

The aim of this study was to evaluate the accuracy of a host of latest wearable devices in measuring fitness-related indicators under various seminatural activities.

**Methods:**

A total of 44 healthy subjects were recruited, and each subject was asked to simultaneously wear 6 devices (Apple Watch 2, Samsung Gear S3, Jawbone Up3, Fitbit Surge, Huawei Talk Band B3, and Xiaomi Mi Band 2) and 2 smartphone apps (Dongdong and Ledongli) to measure five major health indicators (heart rate, number of steps, distance, energy consumption, and sleep duration) under various activity states (resting, walking, running, cycling, and sleeping), which were then compared with the gold standard (manual measurements of the heart rate, number of steps, distance, and sleep, and energy consumption through oxygen consumption) and calculated to determine their respective mean absolute percentage errors (MAPEs).

**Results:**

Wearable devices had a rather high measurement accuracy with respect to heart rate, number of steps, distance, and sleep duration, with a MAPE of approximately 0.10, whereas poor measurement accuracy was observed for energy consumption (calories), indicated by a MAPE of up to 0.44. The measurements varied for the same indicator measured by different fitness trackers. The variation in measurement of the number of steps was the highest (Apple Watch 2: 0.42; Dongdong: 0.01), whereas it was the lowest for heart rate (Samsung Gear S3: 0.34; Xiaomi Mi Band 2: 0.12). Measurements differed insignificantly for the same indicator measured under different states of activity; the MAPE of distance and energy measurements were in the range of 0.08 to 0.17 and 0.41 to 0.48, respectively. Overall, the Samsung Gear S3 performed the best for the measurement of heart rate under the resting state (MAPE of 0.04), whereas Dongdong performed the best for the measurement of the number of steps under the walking state (MAPE of 0.01). Fitbit Surge performed the best for distance measurement under the cycling state (MAPE of 0.04), and Huawei Talk Band B3 performed the best for energy consumption measurement under the walking state (MAPE of 0.17).

**Conclusions:**

At present, mainstream devices are able to reliably measure heart rate, number of steps, distance, and sleep duration, which can be used as effective health evaluation indicators, but the measurement accuracy of energy consumption is still inadequate. Fitness trackers of different brands vary with regard to measurement of indicators and are all affected by the activity state, which indicates that manufacturers of fitness trackers need to improve their algorithms for different activity states.

## Introduction

Level of physical activity (PA) is an important health factor; monitoring and promoting the level of PA can therefore improve people’s health outcomes [1,2]. According to the 2010 World Health Organization (WHO) guideline [3], under normal circumstances, adults aged 18 to 64 years need to engage in at least 150 min of moderate-intensity PA or 75 min of high-intensity PA per week. In addition, to derive more health benefits, 300 min of moderate-intensity PA or 150 min of high-intensity PA per week is required. Currently, large populations in both developed and developing countries have not achieved the recommended levels of PA [2]. For example, 80% of adults in the United States have not reached the recommended level of activity, and around US $117 billion in health care costs are associated with inadequate PA [4]. According to the data released by the WHO in 2014 [5], lack of PA has become the world's fourth greatest mortality risk factor, leading to 3.2 million deaths and 69.3 million lost disability-adjusted life years. The risk of all-cause mortality of adults with low levels of PA is 0.20 to 0.30 higher than that of adults with moderate or high levels of PA. In contrast, increased PA can not only reduce the risk of death of the whole population but also help lower the risk of chronic diseases such as ischemic heart disease, stroke, diabetes, and breast and colon cancers.

Recent years have seen a rapid development of wearable devices such as fitness trackers, which track PA in real time. They are able to enhance users’ PA levels and cultivate healthy living habits because of their superior portability and user-friendly interface and are being accepted by more and more people [6,7]. Compared with the traditional pedometer, fitness trackers are equipped with more accurate sensors and more comprehensive software systems, which not only greatly reduce the discomfort of the wearer but also promote the development of the wearers’ health habits with a friendly interface. Through a systematic review, Bravata et al [8] revealed that fitness tracker use not only increases PA levels but also lowers the user’s body mass index and blood pressure. Poirier et al [9] conducted a randomized controlled trial on 265 individuals, and after 6 weeks of follow-up, they found that in both the mild activity group and the moderate activity group, the number of daily walking steps in the fitness tracker intervention group was significantly higher than that in the control group. Gualtieri et al [10] followed up 10 patients with chronic diseases on fitness tracker use for 3 months and found that the average body weight of the subjects decreased, whereas their PA levels and healthy behaviors improved. After a systematic review, Abedtash et al [11] argued that the combination of fitness tracker and other information technology (IT) measures were more conducive to improving health behaviors. Sullivan et al [12] noted that fitness tracker–based health intervention strategies such as target reminders, progress feedback, healthy behavior recommendations, social encouragement, and other strategies can lead to greater health promotion in the future.

With the development of various miniature sensors, in addition to pedometer functions, wearable device providers are continuously providing new features such as energy consumption measurement, sleep measurement, body temperature measurement, and other feedback. Wen et al [13] surveyed 200 subjects via questionnaire and found that in addition to the aforementioned daily functions, the respondents were more interested in functions more significant to health, such as heart rate monitoring, electrocardiography monitoring, and oxygen saturation monitoring using wearable devices. Therefore, the acquisition of more health-related data through wearable devices will be the focus of future development. Although wearable devices are generally considered as having great potential in health monitoring, the accuracy and reliability of fitness trackers’ monitoring data are the basis and premise on which fitness trackers play their role in health promotion [6,13]. With fitness trackers’ enormous practical value, major technology companies have launched a variety of fitness tracker products, but there’s still a lack of extensive and scientific validation with respect to their accuracy and reliability in health monitoring. Through a systematic review, Evenson et al [14] showed that except for the fact that the measurement of the number of steps is fairly accurate, the measurement of distance overestimates or underestimates with changes in the speed of the activity, and the measurements of energy consumption and sleep duration are usually overestimated relative to the actual values, and the measurements of activity duration varied among different studies; in addition, under different measurement modes, fitness tracker measurements varied significantly. Desilets et al [15] asked 20 subjects to simultaneously wear a fitness tracker and three research-grade heart rate measurement devices and compared the measurement results. They found that when sitting still, the heart rate measurement by the fitness tracker was lower compared with the other devices; under the activity state, the measurements by the fitness tracker and the other devices were also inconsistent, with correlation coefficients (*r*) of .63 to .78. By comparing the accuracies of heart rate and energy consumption measured on 65 subjects under different levels of activity intensity by three types of fitness tracker devices, Dooley et al [16] showed that the measurement accuracies of heart rate measured under all activity states by the Apple Watch 2 and the Garmin Forerunner 225 were high, with the mean absolute percentage error (MAPE) ranging from 0.01 to 0.06, but the measurements of energy consumption were too high, with a MAPE of 0.16 to 0.84. Bai et al [17] asked 52 subjects to complete activities under four intensity levels and compared the measurement accuracies of energy consumption measured by five types of fitness tracker devices and found that the overall error rates of all of the devices ranged from 0.15 to 0.30, which can be even higher in actual use. The results of these studies on the accuracies of wearable devices indicate that various monitoring measurements acquired by wearable devices should be treated precociously.

Given the importance of the accuracy of measurements given by fitness tracker products, the rapid development of the fitness tracker market and the rapid evolution of various brand products, it is necessary to continuously conduct verifications and evaluations of the accuracy of the latest features of the latest products. In this regard, this study has the following characteristics. First, six types of the latest and most representative fitness tracker products, including smart watches, internationally renowned smart bracelets, and smart bracelets popular in China, were included. Second, 2 smartphone apps both having over 50 million users in the Chinese market were included for the first time and compared with fitness tracker devices. Third, the accuracies of the most common and most popular major indicators, including heart rate, number of steps, distance, and energy consumption, under different activity states were simultaneously verified. Fourth, the influences of various activity states were taken into account, and the measurement accuracies of fitness tracker devices under different activity states such as walking, running, and cycling were compared. Fifth, for the first time, the Chinese population was used as the research subject to fill the gap on the Chinese population in such investigations to provide data support for the development of fitness tracker products and a theoretical basis for consumers in choosing products.

## Methods

### Research Equipment and Subject Recruitment

From three types of wearable devices—smart watch, smart bracelet, and smartphone app—8 representative products were chosen. First, when selecting smart watches based on the sales data of Taobao [18] and Jingdong [19], China’s two major electronic commerce platforms, the Apple Watch 2 (Apple Inc, Cupertino, CA, United States) and the Samsung Gear S3 (Samsung Inc, Korea), two top sellers, were chosen as representatives. Second, in selecting smart bracelets, according to the market research data of Canalys and NPD [14-16], Fitbit had the largest market share, and Jawbone had a good market performance and appeared in most of studies. Therefore, the Fitbit Surge (Fitbit Inc, San Francisco, CA, United States) and the Jawbone Up3 (Jawbone Inc, San Francisco, CA, United States) were chosen as representatives of smart bracelets of foreign brands (unfortunately Jawbone was out of business and was no longer producing smart bracelets at the end of this study). In choosing smart bracelets of Chinese brands, the sales of the Xiaomi Mi Band 2 were second only to Fitbit in the health tracking device market, whereas the Huawei Talk Band B2 showed the highest attention among bracelet series products on Zhongguancun Online [20], China’s IT professional website. Therefore, the Xiaomi Mi Band 2 (Mi, China) and the Huawei Talk Band B3 (HUAWEI, China) were chosen as the representatives of smart bracelet of domestic brands. Third, when choosing smartphone apps, according to App Store rankings in the health and fitness category, 2 apps, Dongdong [21] (Dongdong, China) and Ledongli [22] (Ledongli, China) were chosen. The 6 devices and 2 apps were anonymously labeled as FT1-6 and APP1-2 during data analysis so that investigator bias was avoided using a single-blind method.

A total of 44 healthy university students were recruited from Beijing City through open recruitment. The recruitment news was posted in university bulletin boards or forums. The inclusion criteria included 18 years of age, without major illnesses, no allergies to rubber bands, and willing to participate in this study. According to a preset procedure and the product specification, each subject was asked to wear six types of wearable devices to be tested and a standard energy metabolism analyzer to perform the five most common activities. The researchers recorded the corresponding measurements before, during, and after each of the activities. This study was granted permission from the Biomedical Ethics Committee of Peking University, and the subjects were informed on the research objectives and procedures.

### Measurement Indicators and Gold Standard

The five major health indicators—heart rate, number of steps, distance, energy consumption, and sleep duration—which are currently the most common indicators used in fitness tracker monitoring, were used to respectively measure values and gold standards in the states of resting, walking, running, cycling, and sleeping. Manual measurements of heart rate, number of steps, distance, and sleep were used as the gold standards. In measuring energy consumption, a Cosmed K4b2 cardio-pulmonary function tester was used to calculate respiratory quotient and then the energy consumption per unit time [23]. The measurement of sleep duration with the Apple Watch 2 was set as the gold standard because the device requires manual initiation and termination of the sleep mode, and this was the most commonly used method in previous literature.

### Experimental Procedure

Under the resting state, only the indicator of heart rate was measured. First, the subject’s heart rate was measured manually, and then the measurement was repeated using the Apple Watch 2, the Samsung Gear S3, the Fitbit Surge, and the Xiaomi Mi Band 2.

Under the walking state, the subjects were asked to walk on a 400 m standard track for two laps, and the number of steps, distance, and energy consumption were measured. The subjects were asked to wear the Fitbit, the Xiaomi Band 2, and the Apple Watch 2 sequentially from the elbow to the hand on the left wrist and the Samsung, Huawei, and Jawbone bracelets on the right wrist from the elbow to the hand, with the smartphones in the subject’s pocket, while correctly wearing a gas collection device for detecting energy consumption. A researcher followed the subject and recorded the number of steps using a video camera.

Under the state of running, the procedure was identical to that in the case of walking, except that the distance was one lap, and two indicators (distance and energy consumption) were measured.

Under the state of cycling, the subject was asked to ride three trips back and forth in a predetermined route, and the actual cycling distance was recorded with a pedometer（KINGSIR, China）that had been mounted on the bike, while the method of wearing the wearable devices was identical to that in the case of walking. Two indicators (distance and energy consumption) were measured.

Under the state of sleep, the subject was asked to record their going-to-bed and wake-up times with the Apple Watch 2. Duration of sleep was measured.

### Data Management and Analysis

The data acquired by the wearable devices were exported to Excel (Microsoft), saved, and again verified. Heart rate and sleep duration were exported directly, whereas the number of steps, distance, and energy consumption were obtained by extracting the preactivity value from the postactivity value. Outliers derived from the subject’s improper operation were discarded.

Regarding descriptive statistical analysis, the basic information of the subjects, including gender, age, height, weight, and self-stated weekly PA level, was described. MAPE was calculated to reflect the degree of error between the measured value and the true value for each indicator by first dividing the absolute value of the difference between the measured value and the true value with the true value for each sample and then multiplying by 100 and, finally, calculating the mean of all of the samples.

In terms of inferential statistical analysis, Spearman correlation coefficient was employed to evaluate the correlation between the measured value and the true value of each indicator, and the pair-wise *t* test was used to determine whether the difference between the measured value and the true value was statistically significant.

## Results

A total of 44 subjects were enrolled in this study; men and women each accounting for approximately 0.50 of the total. The subjects were aged 19 to 27 years, and the height, weight, and weekly activity levels of the men were significantly higher than those of the women. The basic information of the subjects is shown in Table 1.

Upon comparing the correlation coefficients between the measured and the true values of different indicators, we found that the correlation coefficients between the indicators were statistically significant, indicating that the measured values and the real values were consistent to a certain degree. Among these values, the correlation coefficient of distance was the highest (*r*=.728), whereas that of the number of steps was the lowest (*r*=.342). The pair-wise *t* test result showed that except for the energy consumption measurement (*P*=.19), the differences between the measured and actual values of other indicators were statistically significant (*P*<.05). Details are shown in Table 2.

### Overall Accuracy of the Indicators (After Summarizing the Results of Various Wearable Devices)

Regarding the measurements of heart rate, number of steps, distance, and sleep duration, the accuracies of the wearable devices were fairly high, with a MAPE of approximately 0.10, but the accuracy of the wearable devices for energy consumption (calories) was rather low, with a MAPE of up to 0.44. The differences between the MAPE and SDs of the measurements of different indicators were large, ranging from 0.10 to 0.50. Among the indicators, the measurement of heart rate performed the best, with a MAPE of 0.08 and an SD of error of 0.10, whereas that of energy consumption performed the poorest, with a MAPE of 0.44 and an SD of error of 0.50. Although the measurement accuracies of the number of steps and sleep duration were similar, the measurement of sleep duration was more stable, with an SD of error of 0.17. The MAPE of each indicator is shown in Table 3.

**Table 1:** Basic information about the subjects.

Item	Males (n=23)		Females (n=21)	
	Mean (SD)	Range	Mean (SD)	Range
Age (years)	22.2 (2.2)	19.0-27.0	22.5 (2.1)	19.0-27.0
Height (cm)	173.4 (5.8)	162.0-188.0	161.5 (4.1)	155.0-169.0
Weight (kg)	65.7 (8.9)	51.0-85.0	55.7 (5.0)	47.5-68.0
Weekly physical activity (min)	195.5 (117.7)	60.0-600.0	110 (97.2)	0-360.0

**Table 2:** The correlation coefficients (*r*) and pair-wise *t* test between the measured and the true values of different indicators

Measures	Spearman correlation		Paired sample *t* test
	*r*	*P* value	*P* value
Heart rate	.667	<.001	.045
Step	.342	<.001	<.001
Distance	.728	<.001	<.001
Calorie	.492	<.001	.19
Sleep time	.683	<.001	.007

**Table 3:** Overall accuracies of different measures

Measures	Mean (SD)	Minimum	Maximum	Number
Heart rate	0.08 (0.10)	0.00	0.73	168
Steps	0.09 (0.22)	0.00	0.98	331
Distance	0.13 (0.15)	0.00	0.98	933
Calorie	0.44 (0.50)	0.00	5.79	916
Sleep time	0.11 (0.17)	0.00	1.00	172

### Accuracy of the Same Indicators by Different Wearable Devices

**Figure 1:**
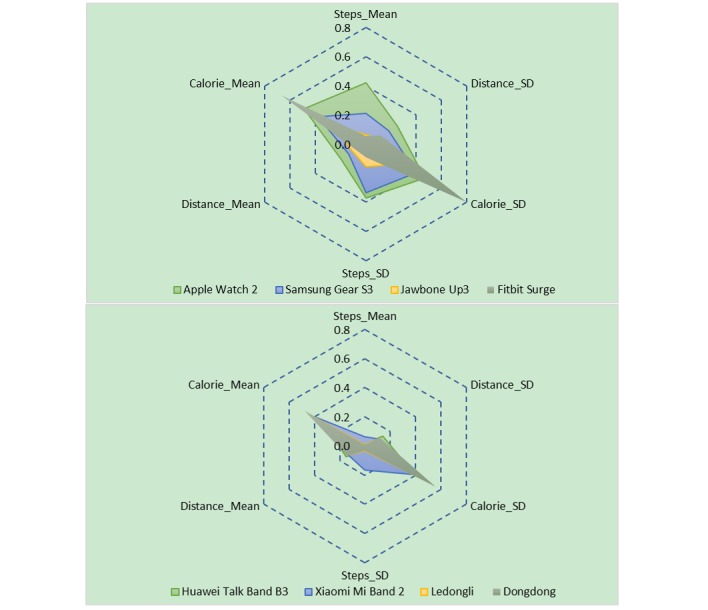
Comprehensive accuracies of indicators by different wearable devices. S=steps, D=distance, and C=calorie.

The radar map of Figure 1 reflects the comprehensive accuracy and stability of the measurement of each indicator by each of the wearable devices, and the results showed that the comprehensive measurement accuracies of different wearable devices differed significantly. Apple Watch 2 and Samsung Gear S3 had a large radar map area and an irregular shape, indicating that their measurement accuracies on different indicators were low and that the stabilities on different indicators were rather poor. Samsung Gear S3 and Jawbone Up3 had a moderate radar map area and a regular shape, indicating that their measurement accuracies on different indicators were appropriate and that the stabilities on different indicators were rather balanced. Fitbit Surge, Huawei Talk Band B3, Xiaomi Mi Band 2, Ledongli, and APP-2 had a small radar map area and a very irregular shape, indicating that their measurement accuracies on energy consumption were low and that their SDs were also rather high; however, their measurement accuracies on distance and the number of steps were high and stable.

Next, we will present the accuracies of each indicator by different wearable devices (see Table 4).

### Heart Rate Accuracy for Different Devices

As shown in Table 4, the subgroup analysis showed that the heart rate measurements recorded with various wearable devices were accurate and stable, whereas the Xiaomi Mi Band 2 performed relatively poorly, with a MAPE of 0.12 and an SD of error of 0.13.

### Step Accuracy for Different Devices

As shown in Table 4, the subgroup analysis showed that the accuracies and stabilities of the wearable devices on the measurement of the number of steps varied significantly, with a lowest MAPE of 0.01 (Dongdong) and a highest MAPE of 0.42 (Apple Watch 2) and with a lowest SD of error of 0.03 (Dongdong and Ledongli) and a highest SD of error of 0.37 (Apple Watch 2). Overall, in terms of the accuracy and stability of the measurement of the number of steps, the Ledongli and the Dongdong gave the best performance, followed by the Huawei Talk Band B3, whereas the Samsung Gear S3 and the Apple Watch 2 performed rather poorly.

### Distance Accuracy for Different Devices

As shown in Table 4, the subgroup analysis showed that except for the Apple Watch 2, the accuracies and stabilities of the various wearable devices on the measurement of distance were similar, with a MAPE of 0.08 to 0.15 and SDs of error of 0.08 to 0.18. The Fitbit Surge had the highest accuracy, whereas the Jawbone Up3 had the highest stability. The MAPE and SD of error of the Apple Watch 2 were 0.20 and 0.25, respectively, having the poorest accuracy and stability.

### Calorie Accuracy for Different Devices

As shown in Table 4, the subgroup analysis on the energy consumption measured by different wearable devices showed that the measurement accuracies and stabilities of all the wearable devices were poor, with a MAPE of 0.28 to 0.67 and SDs of error of 0.27 to 0.80. Of these devices, the Jawbone Up3 gave the best performance, with a MAPE of 0.28 and an SD of error of 0.27, whereas the Fitbit Surge had the worst performance, with a MAPE of 0.67 and an SD of error of 0.80. The other devices performed similarly, with an average MAPE of approximately 0.40 and an SD of approximately 0.40.

### Sleep Time Accuracy for Different Devices

As shown in Table 4, the subgroup analysis on sleep duration measured by the different wearable devices showed that the measurement accuracies and stabilities of all of the wearable devices on sleep duration were good, with a MAPE of 0.06 to 0.17 and SDs of error of 0.10 to 0.21. Of these devices, the Samsung Gear S3 performed the best, and the Huawei Talk Band B3 performed the poorest, whereas the rest performed similarly.

### Accuracy of the Same Indicators Under Different States of Activity

As shown in Table 5, the subgroup analysis on distance measured by different wearable devices under different states of activity showed that under the state of running, the measurements of the wearable devices were both most accurate and most stable, with a MAPE of 0.08 and an SD of error of 0.07. Under the states of walking and cycling, the measurement accuracies of the devices were similar, with a MAPE of 0.16 and 0.17, but the SD of error under the state of walking was far higher than that under the state of cycling, indicating that the measurement of distance in the case of walking was less stable than that in the case of cycling.

As shown in Table 6, the subgroup analysis on energy consumption measured by different wearable devices under different states of activity showed that under different states of activity, the measurement accuracies of the various devices were similar, with a rather high MAPE of over 0.40, whereas the stabilities of the SEs under different states of activity were low and varied remarkably; the SD of error under the state of running was twice as high as that under the state of cycling, being as high as 0.65.

**Table 4:** Accuracies of all indicators by all devices.

Measures/ Device		Mean (SD)	Minimum	Maximum	Number
**Heart rate**					
	Samsung Gear S3	0.04 (0.03)	0.00	0.14	42
	Apple Watch 2	0.07 (0.08)	0.00	0.32	42
	Fitbit Surge	0.08 (0.12)	0.00	0.73	42
	Xiaomi Mi Band 2	0.12 (0.13)	0.00	0.42	42
**Step**					
	Dongdong	0.01 (0.03)	0.00	0.15	44
	Ledongli	0.02 (0.03)	0.00	0.20	44
	Huawei Talk Band B3	0.02 (0.04)	0.00	0.16	44
	Fitbit Surge	0.06 (0.09)	0.00	0.45	44
	Jawbone Up3	0.06 (0.16)	0.00	0.99	44
	Xiaomi Mi Band 2	0.06 (0.17)	0.00	0.96	44
	Samsung Gear S3	0.21 (0.33)	0.00	0.98	39
	Apple Watch 2	0.42 (0.37)	0.00	0.98	29
**Distance**					
	Fitbit Surge	0.08 (0.12)	0.00	0.60	130
	Jawbone Up3	0.10 (0.08)	0.00	0.25	42
	Ledongli	0.12 (0.09)	0.00	0.35	131
	Xiaomi Mi Band 2	0.13 (0.10)	0.00	0.63	131
	Dongdong	0.14 (0.11)	0.00	0.88	130
	Samsung Gear S3	0.14 (0.18)	0.00	0.99	125
	Huawei Talk Band B3	0.15 (0.14)	0.00	0.49	129
	Apple Watch 2	0.20 (0.25)	0.00	0.98	115
**Calorie**					
	Jawbone Up3	0.28 (0.27)	0.00	1.24	43
	Huawei Talk Band B3	0.32 (0.39)	0.00	3.83	128
	Samsung Gear S3	0.38 (0.40)	0.00	4.00	124
	Ledongli	0.39 (0.43)	0.00	3.50	129
	Xiaomi Mi Band 2	0.40 (0.40)	0.00	3.50	128
	Dongdong	0.48 (0.56)	0.00	4.83	128
	Apple Watch 2	0.49 (0.47)	0.00	3.67	125
	Fitbit Surge	0.67 (0.80)	0.00	5.79	111
**Sleep**					
	Samsung Gear S3	0.06 (0.10)	0.00	0.49	43
	Jawbone Up3	0.09 (0.16)	0.00	0.83	43
	Xiaomi Mi Band 2	0.12 (0.19)	0.00	0.87	43
	Huawei Talk Band B3	0.17 (0.21)	0.00	1.00	43

**Table 5:** Distance accuracy for different statuses.

Activity	Mean (SD)	Minimum	Maximum	Number
Walking	0.16 (0.21)	0.00	0.98	323
Running	0.08 (0.07)	0.00	0.55	308
Cycling	0.17 (0.11)	0.00	0.49	302

**Table 6:** Calorie accuracy for different statuses.

Activity	Mean (SD)	Minimum	Maximum	Number
Walking	0.41 (0.50)	0.00	5.79	335
Running	0.42 (0.65)	0.00	4.83	297
Cycling	0.48 (0.30)	0.00	2.20	284

**Figure 2:**
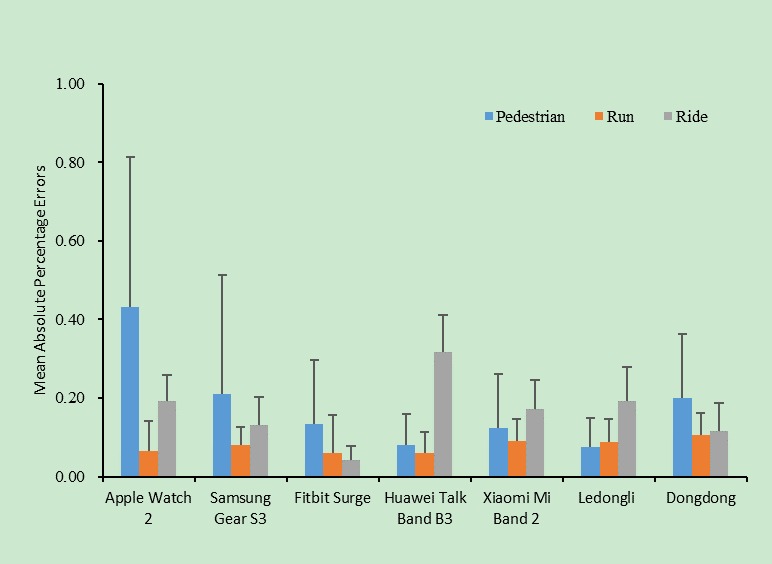
Distance accuracy for different devices used during three different physical activities.

### Accuracy of the Same Indicator by Different Wearable Devices Under Different States of Activity

As shown in Figure 2, the subgroup analysis on distance measured by the different wearable devices under different states of activity showed that except for the Apple Watch 2 and the Huawei Talk Band B3, the measurement accuracies and stabilities of distance under different states of activity varied little. In the case of walking, the MAPE of the Apple Watch 2 was as high as 0.43, whereas in the case of running, it was as low as 0.06.

**Figure 3:**
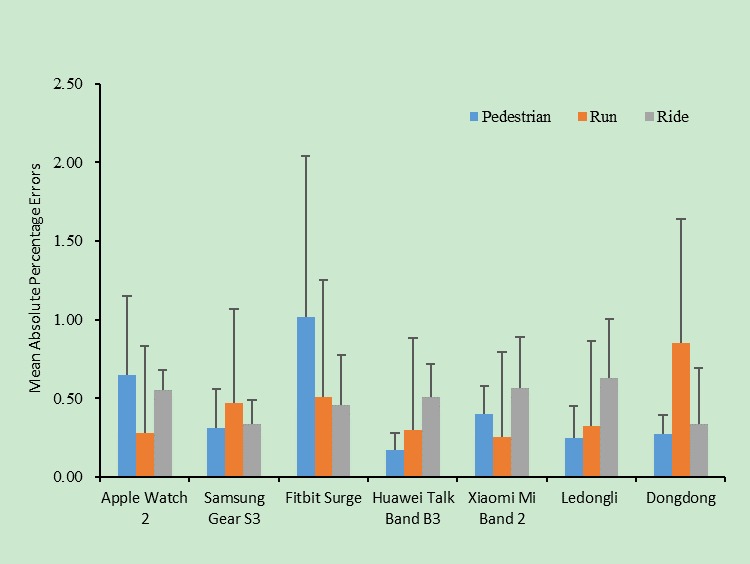
Calorie accuracy for different devices used during three different physical activities.

As shown in Figure 3, the subgroup analysis on distance and energy consumption measured by the different wearable devices under different states of activity showed that the measurement accuracies and stabilities of energy consumption differed significantly. The Fitbit Surge performed the poorest on the measurement of the number of steps but best when used during cycling. The Huawei Talk Band B3 and the Ledongli performed the best on the measurement of the number of steps but poorest when used during cycling. The Apple Watch 2 and the Xiaomi Mi Band 2 performed the best when used while running, whereas the Samsung Gear S3 and the Dongdong performed the poorest when used while running.

## Discussion

### Principal Findings

In this study, we examined the accuracy of the measurements on various data by mainstream wearable devices and mobile apps on the market under seminatural states, and the results showed that in measuring heart rate, number of steps, distance, and sleep duration, the mainstream wearable devices and mobile apps on the market all achieved a rather high accuracy, with MAPE maintained at approximately 0.10. However, in measuring energy consumption, the accuracy was not ideal, and the MAPE was as high as 0.44. In addition, the accuracy on each indicator varied among the individual subjects to varying degrees, with SDs of MAPE ranging from 0.10 to 0.50.

For the measurement of heart rate, Stahl et al [24] examined the measurement accuracies of six types of wearable devices on 50 subjects walking or running on a treadmill and showed that the wearable devices were very accurate in measuring heart rate; the TomTom Runner Cardio performed the best, with a MAPE of only 0.03, whereas the Fitbit Charge HR performed the poorest, with a MAPE of 0.06. Jo et al [25] found that the measurement accuracy of heart rate was significantly affected by activity status; under the state of high PA, the accuracy was significantly reduced. Parak et al [26] found that the accuracies of heart rates measured by different types of wearable devices varied and that the type of sensor and the position in which the device was worn were important factors affecting the accuracy. In this study, we found that the MAPE of a wearable device in measuring heart rate under resting state was approximately 0.08; however, the accuracies of the different wearable devices varied.

For measuring the number of steps, Jones et al [27] evaluated the accuracies of the measurements by 10 types of wearable devices simultaneously worn by 35 subjects under three activity states and found that the measurements were most accurate on the treadmill, with a MAPE of 0.08, followed by that under the state of normal walking, with a MAPE of 0.09, whereas that under the natural state of life was the poorest, with a MAPE of 0.18. Nelson et al [28] found that regardless of the activity state (performing household chores or exercising), the Fitbit models One, Zip, and Flex and the Jawbone UP24 were very accurate in measuring the number of steps, with a MAPE of lower than 0.10. By examining the correlation between the numbers of steps measured by the Fitbit Zip, the ActiGraph GT3X, and the Yamax CW700 pedometers, Mark et al [29] found that the Fitbit Zip was an effective tool for measuring the number of steps taken. Takacs et al [30] examined Fitbit One measurements on 30 adult subjects running on a treadmill at five different speeds and found that the measurement accuracy was affected by the wearer’s running speed. Adam Noah et al [31] showed that under the states of walking and jogging, the Fitbit and the Fitbit Ultra had the highest accuracies in measuring the number of steps. In this study, we found that the devices all had high accuracies in measuring the number of steps, with a MAPE of approximately 0.10, but the MAPE of the Apple Watch 2 was as high as 0.42, likely because of improper operation by the subjects when collecting the data. The Apple Watch 2 requires the watch screen to be awakened so that the data can be correctly synchronized, but in the experiment, the subjects failed in operating it as required, and some of the data were not properly synchronized.

For distance measurements, Takacs et al [30] reported the accuracy of the Fitbit One in measuring distance on a treadmill and that the device had a poor accuracy in measuring distance, with a MAPE of 0.39. In this study, we found that the latest wearable devices have substantially improved the measurement of number of steps, with an average MAPE of 0.14. The subgroup analysis showed that the accuracies of different wearable devices on the measurement of distance were similar, with the best performance when used while running.

For the measurement of energy consumption, John et al [32] evaluated the accuracies of the Sense Wear Armband monitor and the BodyMedia Mini in measuring the energy consumption in the natural state of life over 14 consecutive days and showed that the accuracies of the latest devices were significantly improved compared with those of older models, with the MAPE being reduced to 0.10 and 0.11, respectively. Calabro et al [33] examined the accuracies of three types of wearable devices—the Movband, the Sqord, and the Zamzee—in measuring energy consumption and found that the accuracy was acceptable even for children. Lee et al [34] asked 60 subjects to simultaneously wear eight types of wearable devices in a natural state of life for 69 min and used the Oxycon mobile 5.0 as the gold standard of energy consumption measurement and found that, except for the Basis Band (MAPE=0.23), the majority of the devices had good accuracy, maintaining a MAPE at approximately 0.12. Bonomi et al [35] predicted total energy consumption and energy consumption during activity using the output results of wearable devices and found that the output value of the device and the energy consumption value were clearly correlated. Dannecker et al [36] found that in measuring energy consumption, the accuracy of wearable devices was affected by the activity status; the simpler the activity status, the higher the accuracy. Drenowatz et al [37] subjected 20 subjects to high-intensity PA and found that the accuracy of the Sense Wear Armband monitor in measuring energy consumption under high-intensity PA was much lower than that under low-intensity PA. The results of this study were largely different from those of previous studies, and the MAPE of the Jawbone Up3, which performed the best in measuring energy consumption, was as high as 0.28, and that of the Fitbit Surge, which performed the poorest, was 0.67, far below the acceptable standard.

For the measurement of sleep duration, Montgomery et al [38] found that neither the Fitbit nor the Actigraph was able to accurately identify whether a subject was asleep and often overestimated sleep duration and quality. Lisa et al [39] evaluated the accuracies of the Fitbit Ultra on monitoring sleep with 63 subjects under experimental conditions and showed that the measured value obtained by the Fitbit Ultra and the true value differed significantly. In this study, we found that the wearable devices’ accuracies of sleep duration measurement were rather high, with a MAPE of 0.11, exhibiting little variation among different types of devices.

### Strengths and Limitations of This Study

Compared with previous studies, this study has the following strengths. First, this study simultaneously evaluated multiple latest and most representative wearable devices and mobile phone apps on the market, including internationally renowned smart watches and smart bracelets and smart bracelets of Chinese brands, along with smartphone health apps. Specifically, Dongdong and Ledongli, as the most popular fitness smartphone apps in China, were first studied in our research. These devices are equipped with the latest indicator estimation algorithms, representing the highest level of commercially available wearable devices. Second, the accuracy of the same indicator was examined under different states of activity (resting, walking, running, cycling, and sleeping) so that the measurements were more reasonable and reliable. Third, the evaluation indicators were comprehensive, not only measuring common indicators such as heart rate, number of steps, and distance but also measuring energy consumption and sleep, which essentially cover the most common health monitoring functions of wearable devices. Fourth, this study included 44 subjects who are all college students with high education and little individual variations, had undergone rigorous instruction and training, and were therefore familiar with the operation of various types of devices, thus effectively avoiding the bias derived from improper wearing of the devices. All subjects simultaneously wore different types of fitness trackers so that the selection bias derived from the differences among the individual wearers when comparing different fitness trackers could be avoided. Fifth, the choice of gold standard was reasonable; manual measurements of heart rate, number of steps, distance, and sleep duration were used as the gold standards, which effectively avoided the system error derived from using instrument measurements as gold standards. The calculation of energy consumption through oxygen consumption is currently recognized as the most objective estimation method for energy consumption.

However, the study also has some limitations. First, the monitoring data were acquired from subjects under seminatural circumstances, so the results might not fully reflect those under natural living conditions. For example, in measuring distance while walking, the subjects were asked to walk the preset 800 m, therefore, the accuracy measured under this distance condition may not represent the accuracy under other distance conditions. However, exerting certain restrictions on the subject’s activity conditions can effectively reduce the amount of random and accidental error derived from measurements taken in the natural state of life. Second, although this study included 44 subjects, each indicator under various states of activity was only performed with one cross-section measurement, whereas multiple longitudinal measurements were not conducted, resulting in the reduced size of valid samples. However, the simultaneous measurements on each indicator using a variety of wearable devices compensated for the inadequacy of the sample size to a certain extent. Finally, because of the test conditions and the limitations of the functions of the wearable devices, we did not evaluate the accuracies of various wearable devices in measuring the indicators when the subjects were climbing stairs.

### Conclusions

At present, mainstream devices are able to reliably measure heart rate, number of steps, distance, and sleep duration, which can be used as effective health evaluation indicators, but the measurement accuracy of energy consumption is still inadequate. Fitness trackers of different brands vary with respect to the measurement of indicators and are affected by the activity state. Compared with watch and bracelet, the performance of smartphone apps is better. Future research should further explore why differences among devices exist and how the activity states affect accuracy, thus helping fitness tracker manufacturers improve their algorithms.

## Abbreviations

IT: information technology
MAPE: mean absolute percentage error
PA: physical activity
WHO: World Health Organization
